# Supplementary material for: Immune Microenvironment in Sporadic Early-Onset versus Average-Onset Colorectal Cancer
Source: Cancers (Basel). 2023 Feb 24;15(5):1457. doi: 10.3390/cancers15051457 (PMC10001362; doi:10.3390/cancers15051457)
Supplement: Supplementary file 1 [file cancers-15-01457-s001.zip › cancers-2189210-supplementary.pdf]

Supplementary Materials

Table S1. Antibodies used for immunofluorescence analyses.

Order	Antigen	Clone	Supplier	Dilution	Opal pairing
1	FoxP3	236A/E7	Thermo Fisher	1:250	Opal 520
2	Granzyme B	EPR22645-206	Abcam	1:1000	Opal 570
3	TCRδ	H-41	Santa Cruz Bio-technology	1:1000	Opal 690
4	PanCK	KRT/1877R	Abcam	1:1000	Opal 480
5	CD8α	SP16	Abcam	1:1000	Opal 620
6	CD3	A0452	Agilent Dako	1:250	Opal 780

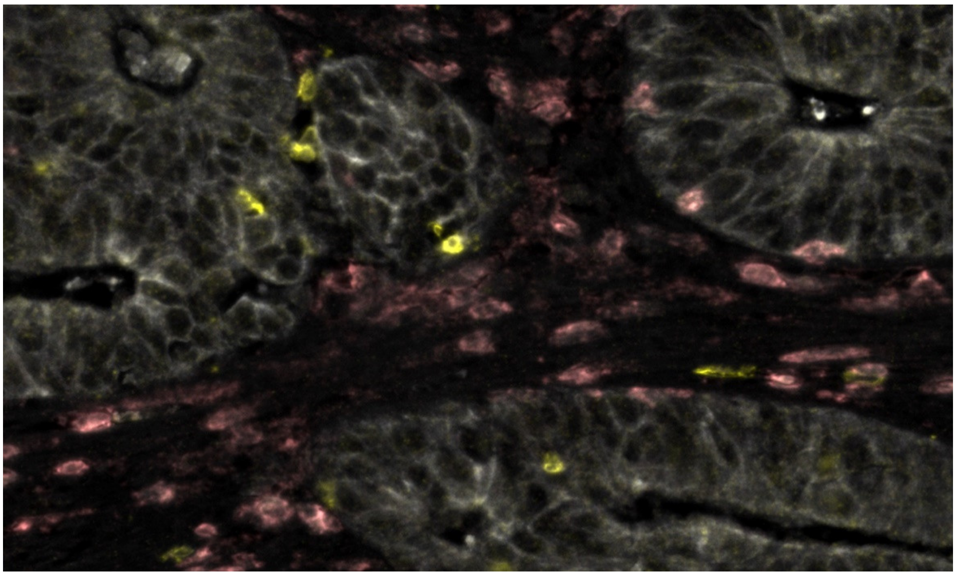

Figure S1. Representative immunofluorescence image with tumor cells (panCK, grey), CD4+ T cells (pink), and CD8+ T cells (yellow).

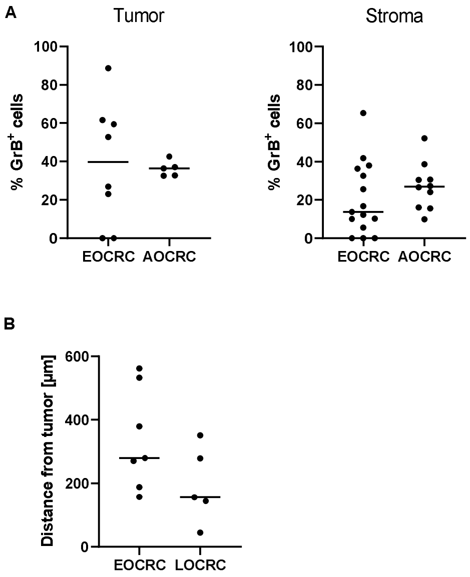

Figure S2. Characteristics of gd T cells in the tumor microenvironment. (**A**) The percentage of gd T cells that express Granzyme B (GrB) in tumor tissue (left panel) and tumor stroma (right panel) was determined by immunofluorescence. (**B**) Mean distance of stromal gd cells to the nearest tumor cell. Symbols show individual values and lines indicate the median.

Table S2. Small NanoString panels of selected genes; Regulatory panel, Cytotoxicity panel and Pro-inflammatory panel.

Regulatory Panel	Cytotoxicity Panel	Pro-Inflammatory Panel
CCR4	CCR5	C3AR1
CD274 (= PD-L1)	CD8A	CCL2
CD4	CD8B	CCL20
CTLA4	CXCR3	CCR6
EBI3 (= IL-35 subunit)	GNLY	CD14
ENTPD1 (= CD39)	GZMA	CD163
FOXP3	GZMB	CD24
ICOS	GZMH	CD80
IDO1	GZMK	CD86
IL-10	GZMM	CR1
IL-12A (= IL-35 subunit)	IFNG	CR2
IL-2RA (= CD25)	KIR3DL1	CXCL1
IL-7R (= CD127)	KIR3DL2	CXCL2
TGFB1	KIR3DL3	CXCL3
TNFRSF18 (= GITR)	KIR_Activating_Subgroup_1	CXCL5
	KIR_Activating_Subgroup_2	CXCL6
	KIR_Inhibiting_Subgroup_1	CXCR1
	KIR_ Inhibiting _Subgroup_2	CXCR2
	KLRB1	ICAM1
	KLRC1	ICAM2
	KLRC2	ICAM3
	KLRD1	IFIT1
	KLRF1	IFIT2
	KLRG1	IFITM1
	KLRK1	IFITM2
	LTA	IFNA1
	LTB	IFNA17
	NCAM1	IFNA2
	PDCD1	IFNA7
	PRF1	IFNA8
	STAT4	IFNB1
	TBX21	IL15
		IL17A
		IL17F
		IL18
		IL1A
		IL1B
		IL22
		IL6
		MYD88
		SELE
		TNF
		VCAM1

Table S3. Methylation detection assay details.

Gene	Assay	Cat No./ID.
MLH1	PyroMark Q24 CpG MLH1 methylation detection assay	970022
MGMT	PyroMark Q24 CpG MGMT methylation detection assay	970032
p16INK4a	Hs_CDKN2A_02_PM PyroMark CpG Assay	PM00039907
LINE-1	PyroMark Q24 CpG LINE-1 methylation detection assay	970042

Pyrosequencing PCR conditions: The PyroMark PCR Kit (Qiagen, Cat No./ID: 978703) was used for the PCR. The Kit includes Coral Load and a PyroMark PCR Master Mix containing HotStarTaq DNA Polymerase, dNTPs, and optimized PyroMark Reaction Buffer with $MgCl_2$. The final $MgCl_2$ concentration was 3 mM for *MLH1*, 2.25 mM for *MGMT* , and 1.5 mM for *p16INK4a* and LINE-1.

Table S4. PyroMark Q24 CpG assays used.

Assay Name	Sequence to Analyze	Number of Cpgs
MLH1	YGGATAGYGATTTTTAAYGYGTAAGYGTATA	5
MGMT	YGTTTTGYGTTTYGAYGTTYGTAGGTTTT	5

p16INK4a	TYGTTAAGTGTTYGGAGTTAATAGTATTTTTTTYGAG-TATTYGTTTAYGGYGT	5[a]
LINE-1	TTYGTGGTGYGTYGTTT	3

PCR conditions for *MLH1 and p16INK4a:* Denaturation at 95 °C for 15 min, followed by 45 cycles of denaturation at 95 °C for 20 s, annealing at 55 °C for 20 s, and extension at 72 °C for 20 s, then final extension at 72 °C for 5 min. PCR conditions for *MGMT:* Denaturation at 95 °C for 15 min, followed by 45 cycles of denaturation at 95 °C for 20 s, annealing at 53 °C for 20 s, and extension at 72 °C for 20 s, then final extension at 72 °C for 5 min. PCR conditions for LINE-1. Denaturation at 95 °C for 15 min, followed by 45 cycles of denaturation at 94 °C for 30 s, annealing at 50 °C for 30 s, and extension at 72 °C for 30 s, then final extension at 72 °C for 10 min. [a] The total number of CpG sites was 6, however, the fifth CpG site position was not included due to a SNP at this site.
